# The North American Layman's Understanding of COVID-19: Are We Doing Enough?

**DOI:** 10.3389/fpubh.2020.00358

**Published:** 2020-07-03

**Authors:** Ali Salimi, Hassan ElHawary, Nermin Diab, Lee Smith

**Affiliations:** ^1^Department of Ophthalmology, Faculty of Medicine, McGill University, Montreal, QC, Canada; ^2^Division of Plastic and Reconstructive Surgery, Faculty of Medicine, McGill University, Montreal, QC, Canada; ^3^Division of Respirology, Faculty of Medicine, University of Toronto, Toronto, ON, Canada; ^4^The Cambridge Centre for Sport and Exercise Sciences, Anglia Ruskin University, Cambridge, United Kingdom

**Keywords:** COVID-19, coronavirus, pandemic, public health, global health, knowledge, risk perception, behavior

## Abstract

**Background:** In the absence of an effective vaccine, public health policies are aimed at awareness, and education of the general public in order to contain the quickly spreading COVID-19 pandemic. Most of the recommended precautionary measures are dependent on human behaviors and therefore their effectiveness largely depends on peoples' perception and attitudes toward the disease. This study aimed to assess the level of knowledge, risk perception, and precautionary measures taken in response to COVID-19 in North America.

**Methods:** In this cross-sectional observational study, an online survey targeted to North Americans focused on the public's knowledge of COVID-19, risk perception, and precautionary behaviors taken in response to this pandemic. Descriptive analyses were performed for the whole population and the subgroup analyses contrasted the differences between Americans and Canadians.

**Results:** The cohort comprised 1,264 relatively young participants with an average age of 28.6 ± 9.8 years. The vast majority (>90%) were knowledgeable about COVID-19. Regarding risk perception, about a quarter assumed to be at less risk to contract the disease, and 42.8% considered themselves to be less contagious than others. While the vast majority avoided performing risky behaviors, only a small proportion (13.2%) wore a face mask**—**which is in line with the public health recommendations of the two countries at the time of data collection. Overall, a larger proportion of Canadian participants (55.8%) were satisfied with the performance of their national public health in response to the current pandemic, compared to their American counterparts (12.2%).

**Discussion:** Data regarding the public's knowledge of COVID-19, risk perception, and behaviors in response to this pandemic is limited. The results of this study highlight that this relatively young and educated sample of North Americans had a high level of knowledge about COVID-19 and a large proportion of them were taking the precautionary measures against this pandemic. However, a significant number of individuals believe to be at less risk of contracting the disease compared to the general population. Educating the public that no one is safe from this disease, could play a role in further limiting risky behaviors and ultimately facilitating disease containment.

## Introduction

What started as an influenza-like virus in Wuhan, China, the SARS-CoV-2 virus and its associated coronavirus disease (COVID-19) has rapidly evolved and been declared a global health emergency by the World Health Organization (WHO) (1). Within several months the virus quickly spread to over 195 counties (2, 3), millions were infected and hundreds of thousands of individuals lost their lives among whom were frontline physicians and healthcare professionals battling against this pandemic (4). Emerging evidence shows that around 80% of individuals who test positive for COVID-19 present with mild respiratory symptoms while almost 14% of cases develop severe-enough symptoms that warrant hospitalization (5). More alarmingly, it has been estimated that around 6% of patients who test positive will experience critical illness and require intensive care admission (5).

While the global incidence rate of COVID-19 is exponentially increasing, different countries have been affected to varying degrees (6). In the absence of an effective vaccine, most countries implemented public health policies that aimed at awareness and containment of the disease (7, 8). However, a recent analysis demonstrated that only half of the countries have strong operational readiness capacities to respond to health emergencies such as COVID-19 (8). Furthermore, different official health agencies recommend different measures to prevent the spread of disease. One example of such differences is the use of face masks for healthy asymptomatic individuals; Early on after the outbreak, China's national recommendations included wearing face masks for both health-care professionals as well as the general public while the United States'(US) Surgeon General advised against using face masks for asymptomatic patients citing the absence of strong evidence against COVID-19 infection (9). Over time, these recommendations have been subject to change, as more is discovered about the virus (10).

Many of the precautionary and preventative measures taken in different countries are dependent on human behaviors and therefore public health response effectiveness largely depends on peoples' perception of the disease and their attitude toward it (11). Previous studies have shown significant differences between Europeans and Asians' attitudes and risk perceptions of the 2003 Severe Acute Respiratory Syndrome (SARS) outbreak (12). Similar differences have been evidenced between countries with relatively similar cultures and close geographic location (13).

To that end, this study aimed to compare and contrast the level of knowledge, risk perception, and precautionary measures taken in response to COVID-19, between populations of the United States of America (US) and Canada. To date, the US has reported the highest rate of COVID-19 positive cases in the world and therefore, by understanding the public's attitude and risk perception toward the current pandemic, we hope to provide valuable information to help develop adequate population-tailored communication protocols that are effective in disease prevention and containment.

## Methods

This cross-sectional observational study reports on unique aspects of knowledge, risk perception, and precautionary behaviors related to COVID-19, among a large sample of North Americans. Voluntary informed consent was presented on the front page of the questionnaire and was electronically signed by all participants before gaining access to the questions. The study was approved by the institutional review board of McGill University, Montreal, Canada.

### Questionnaire and Recruitment

The questionnaire was developed on a secure and encrypted cloud-based database and was adapted from similar previous studies on severe acute respiratory syndrome (SARS) and the Middle East respiratory syndrome (MERS) (13–15). It entailed a total of 34 questions including demographics, knowledge of COVID-19 along with sources from which information was gained and confidence in each, perceived risks of virus contraction and dissemination, as well as changes in individual behaviors following the onset of this pandemic (Appendix 1). Response to all questions was required for completion and submission of the questionnaire, and incomplete answers were not registered in the database. Response rate was the ratio of the completed questionnaires to the total number of individuals who accessed the questionnaire (whether completed or not). To prevent duplicate answers a cookie-based protection system was used.

Demographic information included age, sex, health status, level of education, self-reported income as a proxy for socioeconomic status (SES), country of residence, and living status. The knowledge questions focused on the self-reported understanding of the disease, etiology, mode of transmission, and phase(s) of contagiousness. Risk perception assessed the anticipated likelihood of catching a common cold, getting a heart attack, or contracting COVID-19; self-perceived risk of contracting, and disseminating COVID-19 compared to the general population; confidence in the ability to avoid contracting the disease; and worry about their health as well as that of their loved ones.

Behavioral questions assessed abstinence from performing risky behaviors such as traveling, leaving the house, eating outdoors, shaking hands, using public transportation, participating in large gatherings, and touching one's face**—**more specifically, the eyes, nose, and mouth. Additionally, frequent hand washing, and undertaking lifestyle changes including better sleep, a more balanced diet, and exercising, were assessed.

Data collection occurred in March 2020. The questionnaire was disseminated through social media (Facebook, LinkedIn, Research Gate, Instagram, and Twitter) and a variety of web-based platforms and forums such as surverycircle.com and surveyswap.io targeting Americans and Canadians. The data were only accessible to the lead authors, and to maintain the full anonymity of participants no personally identifiable information was obtained.

### Statistical Analysis

The Shapiro-Wilk test verified that the continuous data respected the parameters for normality. Descriptive analyses were performed for the whole population and the repeated measures Analysis of Variance (ANOVAs) assessed differences in reliance and confidence in available information sources concerning the COVID-19. Data were analyzed for Canada and the United States (US) separately and were compared using Pearson's chi-square (categorical outcomes) and General Linear Models (continuous outcomes), accounting for any demographic differences across the two groups. Given the online nature of the survey**—**which typically attracts younger individuals with better technological skills, supplementary analyses including stratification by age and the level of education were performed using Student's t-test (for continuous variables) and Pearson's chi-square test (for categorical outcomes). Participants less than 40 years old were considered as younger adults while those older than 40 years were included in the middle-aged and older adults group (16). Stratification by the level of education consisted of individuals with a high school diploma or less versus those with a minimum of a bachelor's degree. All statistical analyses were performed using SPSS 25.0 (IBM, New York, USA) with significance set at p < 0.05.

## Results

### Demographics

The survey was accessed by a total of 1,731 individuals, among which 1,264 completed the survey by answering all questions**—**a response rate of 73.5%. Out of the 1,264 participants, 913 (72%) were from the US, and 351 (28%) were from Canada. The cohort consisted of 728 females and 536 males, with an average age of 28.6 ± 9.8 years. The majority (64%) cohabited with healthy individuals, 22% resided with vulnerable populations (immunocompromised, elderly, or children), and 14% lived alone. Chronic disease was reported among 18%. The majority of the participants had obtained a university diploma (68%) and the remaining 32% had a high school degree or less. Two-thirds of the participants were employed, and the remaining one-third were either students, stay-home parents, or retired. SES was reported as low or middle-low among 30%, middle among 40%, and high or middle-high among the remaining 30%.

None of the demographic measures were different across the two groups, except the education level where a larger proportion of Canadians had a university degree (χ^2^ = 13.160; *p* = 0.003) (Table 1).

**Table 1:** Demographic characteristics.

**Characteristics**	**Whole cohort (*n* = 1,264)**	**Americans (*n* = 913)**	**Canadians (*n* = 351)**	**95% confidence interval**	***p*-value**
Age (years)	28.64 ± 9.76	28.9 ± 10.0	28.0 ± 9.0	−0.339 – 2.064	0.139
	[18–79]	[18–75]	[18–79]		
**Characteristics**	**Whole cohort (*****n*** **=** **1,264)**	**Americans (*****n*** **=** **913)**	**Canadians (*****n*** **=** **351)**	**Chi-square value (*****χ***^**2**^**)**	***p*****-value**
Sex (M:F)				0.130	0.718
Male	536 (42.4%)	390 (42.7%)	146 (41.6%)		
Female	728 (57.6%)	523 (57.3%)	205 (58.4%)		
Living status				2.797	0.247
Alone	176 (13.9%)	124 (13.6%)	52 (14.8%)		
With healthy individuals	811 (64.2%)	578 (63.3%)	233 (66.4%)		
With vulnerable individuals	277 (21.9%)	211 (23.1%)	66 (18.8%)		
Comorbidity	233 (18.4%)	180 (19.7%)	53 (15.1%)	3.592	0.063
Education				13.160	0.005^*^
Less than high school	91 (7.2%)	77 (8.4%)	14 (4.0%)		
High school diploma	315 (24.9%)	240 (26.3%)	75 (21.4%)		
Bachelors or vocational degree	577 (45.6%)	405 (44.4%)	172 (49.0%)		
Graduate or professional degree	281 (22.2%)	191 (20.9%)	90 (25.6%)		
Socioeconomic class				3.212	0.523
Low	88 (7.0%)	67 (7.3%)	21 (6.0%)		
Middle-low	289 (22.9%)	212 (23.2%)	77 (21.9%)		
Middle	512 (40.5%)	363 (39.8%)	149 (42.5%)		
Middle-high	328 (25.9%)	241 (26.4%)	87 (24.8%)		
High	47 (3.7%)	30 (3.3%)	17 (4.8%)		

* *Denotes statistically significant differences between Americans and Canadians; ± represents mean ± standard deviation; [] represents the range*.

### Knowledge of COVID-19

Self-reported knowledge of COVID-19, measured on a 5-point Likert scale, was averaged to 3.72 ± 0.77 (out of five). The cause of COVID-19 was correctly identified to be a virus by 98.7% and the average mortality rate was reported by the participants to be 3.95 ± 5.69%. Respiratory droplets were recognized as the most common mode of transmission by the majority of the participants (90.1%), followed by airborne (9.3%), and feco-oral (0.6%). Intergroup analyses highlighted that a significantly larger proportion of Canadians (94%) identified droplets as the correct answer compared to Americans (88.6%) (χ^2^ = 9.365; *p* = 0.009). The absence of any commercially available vaccine against COVID-19 (which was the case at the time of the study) was correctly recognized by 98.2%. In terms of contagiousness of COVID-19, 92.1% were aware that the virus can spread during both asymptomatic and symptomatic phases, as opposed to only during the asymptomatic phase (7%) or only during the symptomatic phase (0.9%).

Participants reported gaining the majority of their COVID-19 information from official health agency channels (4.00 ± 1.01, out of five), which was significantly higher (*F*-value = 370.998; *p* < 0.001) than the alternative information sources: printed or online version of newspapers and magazines (3.13 ± 1.35), social media (2.70 ± 1.30), friends or relatives (2.31 ± 1.04), and television (1.93 ± 1.14). Similarly, confidence in information released by public health authorities was 4.44 ± 0.82 (out of five), significantly higher (*F*-value = 2350.686; *p* < 0.001) than printed or online newspaper and magazines (2.90 ± 1.05), friends or relatives (2.15 ± 0.87), television (2.13 ± 0.99), and social media (1.71 ± 0.82). No statistically significant differences were found between the American and Canadian participants with regards to their information sources or the confidence in each source (Table 2).

**Table 2:** Source of information about COVID-19 and confidence in each source (*n* = 1,264).

**Information source**	**Amount of info gained**	**Confidence in the source**
Official health agency channels	4.00 ± 1.01	4.44 ± 0.82
Printed or online newspapers or magazines	3.13 ± 1.35	2.90 ± 1.05
Social media	2.70 ± 1.30	2.15 ± 0.87
Friends or relatives	2.31 ± 1.04	2.13 ± 0.99
Television	1.93 ± 1.14	1.71 ± 0.82

*All variables were measured on a 5-point Likert scale*.

*± represents mean ± standard deviation*.

### Risk Perception of COVID-19

On average, both groups of participants were significantly more concerned for the health of their loved ones (4.18 ± 0.98, out of five) over that of their own (2.97 ± 1.22; 95% CI = 1.145–1.271; *p* < 0.001).

Participants rated the likelihood of contracting COVID-19 during this pandemic to be 3.24 ± 1.01 (out of five), which was significantly less likely than catching a common cold (4.71 ± 0.71; 95% CI = 0.313–0.449; *p* < 0.001) but significantly more likely than getting a heart attack in their lifetime (2.86 ± 0.90; 95% CI = 1.405–1.531; *p* < 0.001). No significant differences were found between the two groups.

While over half of the participants (58.2%) believed they have the same risk of contracting COVID-19 as the rest of the population, a quarter (25.8%) believed they are at less risk, while 16% believed to be at more risk compared to the general population. Interestingly, the perceived degree of contagiousness was rated to be 1.66 ± 0.91 (out of five). Moreover, 42.8% of the participants considered themselves to be less contagious than others with only a minority (5.5%) believing to be more contagious than the general population. About half of the population (49.4%) believed that they can avoid contracting COVID-19, while the other half (50.6%) were unsure or believed otherwise.

### Precautionary Behaviors

The results show that jobs were notably affected by the current pandemic; among those employed, over half (53.5%) had transitioned to working remotely from home, 22.6% had stopped working, while only 23.9% were still physically going to work. This impact was larger on Canadians, as a larger proportion (83.1%) had stopped working or transitioned to working online (vs. 72.8 % Americans) and a smaller proportion (16.9%) were still physically going to work (vs. 27.2% Americans) (χ^2^ = 21.988; *p* < 0.001).

Abstinence from risky behaviors was reported by a large proportion of the participants, including avoiding traveling (97.0%), leaving the house (95.0%), eating outdoors (97.2%), shaking hands (97.2%), using public transportation (95.9%), participating in large gatherings (98.6%), and touching their face (69.0%). Comparisons between the two groups highlighted that a larger proportion of Canadian participants abstained from traveling (99.4 vs. 96.1% Americans; χ^2^ = 9.893; *p* = 0.008), and avoided touching their face (81.2% vs. 64.3 Americans; χ^2^ = 33.857; *p* < 0.001).

Frequent hand washing was reported by 95.3% of the participants, and regular use of disinfectants to clean surfaces at home was described by 62.7%. On average, wearing a mask outdoors was reported by only 13.2%, which was significantly higher among American participants (14.5%) compared to their Canadian counterparts (10.0%; χ^2^ = 4.450; *p* = 0.028).

Life-style changes in response to this pandemic included healthier sleep habits (62.7%), a more balanced diet (59.3%), and exercising (52.1%). The latter two were more prominent amongst Canadian participants, as a larger proportion implemented a healthier diet (67.2 vs. 56.2% Americans; χ^2^ = 12.818; *p* < 0.001) and exercising (62.4 vs. 48.1% Americans; χ^2^ = 20.802; *p* < 0.001).

The participants were asked about their presumed behavior under a hypothetical assumption where they have been suspected of being a COVID-19 carrier and are recommended to remain in self-isolation; under this assumption, the majority (96.8%) reported to conform to the recommendation and remain in complete self-isolation, 2.3% stated that would obey the recommendation but would prioritize personal affairs, and 0.9% reported to refuse isolation and leave the house. The comparison between the two groups highlighted that a larger proportion of Canadian participants (99.4%) would conform with the isolation recommendations if they were to become suspected of being a COVID-19 carrier (compared to 95.8% Americans; χ^2^ = 10.677; *p* = 0.001)

Finally, the overall satisfaction with regards to the national public health response to COVID-19 was significantly higher among Canadians. Over half of the Canadian participants (55.8%) believed that the national public health response was sufficient or adequate while only 12.2% of the American participants shared a similar belief about the US public health response (χ^2^ = 263.084; *p* < 0.001).

#### Supplementary Analyses Stratified by Age

With a cut-off age of 40 years, the younger adults group consisted of 1,118 individuals with an average age of 25.9 ± 5.8 years vs. the middle-aged and the middle-aged and older adults group (referred to older adults group herein, for simplicity) included 146 individuals with an average age of 49.5 ± 8.4 years. Differences in demographics, in addition to the age (95% CI = 22.551–24.687; *p* < 0.001), included more individuals in the older group with a higher level of education (76 vs. 67% in the younger group; χ^2^ = 5.026; *p* = 0.025), living with immune-compromised individuals (36 vs. 20% in the younger group; χ^2^ = 27.309; *p* < 0.001), and having comorbidities (45 vs. 15% in the younger group; χ^2^ = 74.711; *p* < 0.001).

Self-reported knowledge of COVID-19, measured on a 5-point Likert scale, was significantly higher among the older adults (4.09 ± 0.76) compared to younger adults (3.67 ± 0.76; 95% CI = 0.282–0.544; *p* < 0.001). Older adults, compared to their younger counterparts, relied more on television as a source of information (2.2 ± 1.3 vs. 1.9 ± 1.1; 95% CI = 0.102–0.495; *p* = 0.008) and less on social media (2.4 ± 1.3 vs. 2.7 ± 1.3; 95%CI = 0.099–0.545; *p* = 0.005).

Older adults, when compared to their younger counterparts, were more concerned for their own health (3.64 ± 1.09 vs. 2.88 ± 1.21; 95%CI = 0.548–0.962; *p* < 0.001); though, both groups were equally concerned for the health of their loved ones (*p* > 0.05). The perceived risk of disease contraction and the degree of contagiousness was not different between the two groups (*p* > 0.05).

There were no differences between the two groups in terms of taking precautionary actions and abstaining from risky behaviors, except for face-mask-wearing which was reported among a larger proportion of older adults (21% compared to 12% in younger adults; χ^2^ = 7.747; *p* = 0.005). Interestingly, a larger proportion of older adults reported to exercise (61 vs. 51% among young adults; χ^2^ = 5.241; *p* = 0.022) and to keep a balanced diet (73 vs. 57%; χ^2^ = 13.461; *p* < 0.001), in response to COVID-19. Satisfaction with public health response was similar among the two groups (χ^2^ = 0.255; *p* = 0.682).

#### Supplementary Analysis Stratified by the Level of Education

The less educated group (high school diploma or less) consisted of 406 individuals with an average age of 24.6 ± 9.9 years vs. the more educated group (a minimum of bachelor's degree or equivalent) which included 858 individuals with an average age of 30.6 ± 9.1 years. In terms of the demographic differences, in addition to the younger age among the less educated group (95% CI = 4.886–7.097; *p* < 0.001), a larger proportion of them had lower SES (39 vs. 25% in the more educated group; χ^2^ = 27.958; *p* < 0.001), and a smaller proportion were living alone (7 vs. 17% in the more educated group; χ^2^ = 27.344; *p* < 0.001).

In terms of knowledge, a larger proportion of those with lower education wrongly identified bacteria as the cause of COVID-19 (2.5 vs. 0.8% in the more educated group; χ^2^ = 5.636; *p* = 0.018) and associated the main mode of transmission to airborne (12 vs. 8% in the more educated group; χ^2^ = 9.589; *p* = 0.008). Those with a lower level of education relied more on social media as a source of information (2.9 ± 1.3 vs. 2.6 ± 1.3 in the more educated group; 95%CI = 0.152–0.456; *p* < 0.001) and less on official public health sources (3.9 ± 1.1 vs. 4.1 ± 1.0 in the more educated group; 95%CI = 0.032–0.270; *p* = 0.013).

In terms of risk perception, a larger proportion of those with lower education thought to be at lower risk of disease contraction (33 vs. 22% in the more educated group; χ^2^ = 19.415; *p* < 0.001) and to be less contagious relative to the general population (49 vs. 40%; χ^2^ = 8.040; *p* = 0.018).

With regards to the precautionary actions against COVID-19, a larger proportion of those with lower education reported risky behaviors such leaving the house (8 vs. 3% in the higher educated group; χ^2^ = 12.483; *p* < 0.001), shaking hands (4 vs. 2% in the higher educated group; χ^2^ = 5.433; *p* = 0.020), participating in gatherings (3 vs. 1% in the higher educated group; χ^2^ = 4.600; *p* = 0.032), and touching their face outdoors (40 vs. 26% in the higher educated group; χ^2^ = 23.330; *p* < 0.001). Frequent handwashing was reported among a smaller proportion of those with lower education (92 vs. 97% in the higher educated group; χ^2^ = 9.955; *p* = 0.002). A larger proportion of those with higher education reported adopting healthier sleeping habits (67 vs. 53% in the lower educated group; χ^2^ = 22.076; *p* < 0.001), exercising (56 vs. 42% in the lower educated group; χ^2^ = 20.283; *p* < 0.001), and keeping a balanced diet (65 vs. 47% in the lower educated group; χ^2^ = 36.946; *p* < 0.001), in response to COVID-19. No differences were found between the two groups in terms of face-mask-wearing (χ^2^ = 1.396, *p* = 0.237) or satisfaction with public health response (χ^2^ = 2.221; *p* = 0.136).

## Discussion

To the best of authors' knowledge, this paper represents the first study to assess perception and attitudes toward the COVID-19 pandemic, among a large cohort of individuals in North America. Our results show that this sample of relatively young and educated American and Canadian participants had a high level of subjective and objective knowledge of the COVID-19 disease. However, over a quarter of the sample believed they were less likely to contract the disease compared to others, and more interestingly, almost half of them considered themselves to be less contagious than the general population. That being said, both Canadians and Americans participants reported avoiding many risky behaviors associated with the COVID-19 spread such as, participating in large gatherings, shaking hands, eating outside, and using public transportation, and they mostly engaged in healthy habits such as frequent hand washing. Canadians were more likely to avoid touching their face, implement a healthier diet, and exercise, compared to Americans. Both populations reported to not frequently use face masks**—**which was in accordance with the public health recommendations of the two countries at the time**—**with Americans using them slightly more than Canadians.

One of the public health priorities during pandemics is to influence the general public's attitudes and perceptions (17). This notion of public perception becomes vitally important during pandemics such as COVID-19 where a vaccine or effective treatment is not available. There is a prevailing view that the general public was resistant to public health recommendations during pandemics such as H1N1 influenza and SARS outbreaks (17). While the findings of this study cannot be directly related to public health performance, it sheds light on the knowledge and perception of COVID-19 among a relatively young and educated sample in North America. Our results show that both groups have a very accurate understanding of the SARS-CoV-2 virus including the methods of transmission and the average mortality rate. This contrasts with findings of some of the SARS pandemic studies that found that almost half of the people thought SARS was curable early on during the pandemic (compared to only 1.8% of our sample who believed that there is a cure or vaccine for COVID-19) (18). Similarly, several previous studies showed that the public perceived their risk of contracting SARS to be quite low, a finding that contrasts with our results in the context of the COVID-19 pandemic (14, 18). These differences are likely multifactorial. Differences in age, ethnicity, and level of education of the samples could explain some of the differences in the findings. For instance, our sample constituted of relatively young and educated participants that had access to the internet and were willing to participate in our online study in contrast to Lau et al. study, where age and education were more evenly distributed (18). Other reasons include the timing and the era in which the study was conducted. Living in the era of technology and abundance of information facilitates access to the information; hence, comparing the public knowledge about a pandemic today and contrasting it to that of 17 years prior could be erroneous. Further, our study was conducted at least 90 days after the disease outbreak compared to that of Lau et al., which was conducted from day 11 to 60 of the SARS outbreak, leaving the public with limited time to inform themselves of the reality of their time. Lastly, while we cannot make a direct association, the role of prompt public health response in the US and Canada based on the lessons learned from previous pandemics cannot be ruled out.

A notable proportion believed they are at less risk of contracting COVID-19 and are less contagious than the general public (25.8 and 42.8%, respectively). A possible explanation to these findings could be related to the relatively young average age of our study participants (28.6 ± 9.8 years) and the widespread perception that younger individuals are unlikely to be affected by COVID-19 compared to older individuals (19). One can mitigate the risk of contracting the disease, and hence be less contagious, by taking the necessary precautionary measures; however, this idea that adverse outcomes only happen to others stems from the human psyche. Previous studies across different domains have shown that people generally tend to be optimistic regarding their health and usually underestimate their own risk of suffering from diseases or health-related negative outcomes (20, 21). This could be one of the explanations for why almost half of our sample believed they were able to avoid contracting COVID-19. While being optimistic and having a sense of internal locus of control are not necessarily undesirable, they can both lead to less caution and riskier behaviors among individuals and ultimately contribute to the spread of the disease.

Our findings show that both American and Canadian participants gain most of their COVID-19 health-related knowledge from official health agencies. Similarly, they had higher confidence in the knowledge gained from these sources compared to other sources. This could be explained by the fact the majority of both Canadian and American participants in our sample was relatively well-educated and therefore were more likely to have confidence in more trusted sources such as official public health agencies. This hypothesis can be supported by our sub-analysis that showed that the participants with a lower level of education (high school diploma or less) relied more social media and less on public health resources to gain their COVID-19-related information. Finally, it is important to acknowledge that public health information is often linked with political information and while the questionnaire included clear examples of what constitutes a public health source of information, it is often difficult for the public to differentiate them and therefore our results should be interpreted in this context.

Both the Center for Disease Control and Prevention (CDC) and Health Canada published several precautionary measures to be taken by the general population including frequent hand washing, practicing social isolation, and avoiding gatherings, or unnecessary travel (10, 22). Our data highlights that the majority of our sample were taking many of these precautionary measures. However, while both American and Canadian official health agencies recommend against touching one's eyes, nose, or mouth, a significantly larger portion of Canadians follow that recommendation compared to their American counterparts. This could be partially explained by the fact that more Canadian participants correctly identified that the SARA-CoV-2 virus is transmitted through respiratory droplets. Moreover, more Canadian participants implemented a healthier diet and exercised to help prevent contracting COVID-19; recommendations suggested by the WHO (23). While these findings are quite positive, they must not be overgeneralized. Our sample consisted of relatively young and educated participants and therefore the findings might not apply to the general population. Our sub-analysis showed that a smaller proportion of those with a lower educational level engaged in such lifestyle changes.

The majority of the participants in both groups did not wear face masks which could be attributed to the recommendations set by both official health agencies against mask-wearing among the asymptomatic individuals at the time of the conduction of the study. It is important to note that both Canadian and American official healthcare guidelines changed following data collection, in favor of wearing face masks due to the strong evidence supporting its benefits (24–26). It would be interesting for future studies to re-assess the population's perceptions and behaviors regarding wearing facial masks after this change in recommendations.

The study's main limitation is its selection bias and that the majority of the participants are young and highly educated, in line with the online nature of the data collection. This limits the generalizability of the results and thereby the findings should be interpreted within the context of this limitation. In an attempt to mitigate this limitation to certain extents, a sub-analysis of data stratified by age and level of education was performed; nevertheless, the limitation still exists. Moreover, the study only included North Americans. As previous studies show, people from different regions and cultures have different attitudes and risk perceptions of pandemics (14, 18). Therefore future works should study the level of knowledge, risk perception, and precautionary behaviors taken by individuals in different regions. Our study did not assess the differences in attitudes and perceptions across different states and provinces of each country; given to the large and heterogeneous multi-cultural populations of the US and Canada, future studies should assess any differences in these factors. Finally, we encourage future studies to study the types of messages that are being received from health sources and to compare this between the two countries. This will help provide useful information for future health care emergencies and pandemics.

## Conclusion

The COVID-19 pandemic represents a global health emergency that has affected virtually all countries. Due to the rapidly rising incidence rate in North America coupled with the lack of available vaccination or cure, public health response along with people's attitudes toward this pandemic is of paramount importance in preventing the spread of disease. The present study highlights a high level of knowledge about COVID-19 and good precautionary measures taken against this pandemic, among relatively young and educated North American participants. However, with many young individuals believing to be less at risk or able to avoid contracting the COVID-19, the authors believe that more resources should be invested in educating the public that no one is safe from this disease and therefore everyone should continue taking maximum precautionary measures until the disease is contained.

## Data Availability Statement

The raw data supporting the conclusions of this article will be made available by the authors, without undue reservation.

## Ethics Statement

This study was approved by the Institutional Review Board of McGill University (Montreal, Canada). Voluntary informed consent was presented on the front page of the questionnaire and was electronically signed by all participants before gaining access to the questions.

## Author Contributions

AS and HE substantially contributed to the conception and design, data acquisition, analysis and interpretation of data, and drafting of the manuscript. ND and LS contributed to the design, data interpretation, and revised the manuscript critically. All authors agree to be accountable for all aspects of the work.

## Conflict of Interest

The authors declare that the research was conducted in the absence of any commercial or financial relationships that could be construed as a potential conflict of interest.
