# Supplementary material for: The North American Layman's Understanding of COVID-19: Are We Doing Enough?
Source: Front Public Health. 2020 Jul 3;8:358. doi: 10.3389/fpubh.2020.00358 (PMC7349001; doi:10.3389/fpubh.2020.00358)
Supplement: Supplementary file 1 [file Data_Sheet_1.pdf]

The North American Layman's Understanding of COVID-19: Are We Doing Enough?

Ali Salimi, Hassan Elhawary, Nermin Diab, Lee Smith

Appendix 1. Survey questions.

1) Age
2) Gender
3) What is your highest education degree attained
    a. Have not completed high school
    b. High school
    c. Bachelor's or vocational degree
    d. Post-graduate or professional degree
4) Socioeconomic status
    a. Low
    b. Low/middle
    c. Middle
    d. Middle/High
    e. High
5) What country do you live in?
    a. United States of America
    b. Canada
6) Living status?
    a. Alone
    b. With others (healthy)
    c. With others (immunocompromised, elderly, kids)
7) Do you have any chronic conditions that makes you vulnerable to infections such as COVID-19?
    a. Yes
    b. No
8) Has you work been affected by COVID-19
    a. No; I am still physically going to work
    b. Yes; I am working from home
    c. Yes; I am NOT working due to COVID-19
    d. I was unemployed prior to COVID-19 pandemic

(Salimi et al., 2020)

9) How would you rate your level of knowledge of COVID-19 (1 very poor ←→5 Excellent)
10) What causes COVID-19?
    a. Virus
    b. Bacteria
    c. Fungus
    d. Parasite
11) How is SARS-CoV-2 (the organism responsible for COVID-19) transmitted
    a. Airborne
    b. Respiratory droplets
    c. Fecal oral
    d. Insect bites
12) When do you think a person is contagious?
    a. Prior to any symptoms
    b. Only during symptoms
    c. Both prior and during symptoms
13) Is there currently a commercially available vaccine for COVID-19?
    a. Yes
    b. No
14) In your lifetime, what is the likelihood of you contracting Flu or common cold
    a. (1 very unlikely ←→ 5 very likely)
15) In your lifetime, what is the likelihood of you contracting Heart attack
    a. (1 very unlikely ←→ 5 very likely)
16) In your lifetime, what is the likelihood of you contracting COVID-19
    a. (1 very unlikely ←→ 5 very likely)
17) What is your risk of contracting COVID-19 compared to the general population?
    a. Less than the general population
    b. Same as the general
    c. Higher than the general population
18) How contagious do you think you currently are compared to the general population?
    a. Less than the general population
    b. Same as the general population
    c. More than the general population

(Salimi et al., 2020)

19) What is the average death rate of COVID-19 (in %)

20) Do you think you can avoid contracting COVID-19

a. Yes

b. No

21) Given the current COVID-19 pandemic, how worried are you about

a. your own health (1 Not worried ←→ 5 very worried)

b. your family/loved one's health (1 Not worried ←→ 5 very worried)

22) What behaviours have you done to prevent COVID-19 infection?

a. Avoiding travel (Yes/No)

b. Avoiding leaving your house for unnecessary affairs (Yes/No)

c. Avoiding eating in restaurants (Yes/No)

d. Avoiding shaking hands (Yes/No)

e. Avoiding using public transportation (Yes/No)

f. Avoiding large gatherings (Yes/No)

g. Limiting touching your face (as much as possible) (Yes/No)

h. Washing your hands more often (Yes/No)

i. Using disinfectants to clean your house more often (Yes/No)

j. Wearing a mask outdoors (Yes/No)

k. Sleeping well (Yes/No)

l. Eating a balanced diet (Yes/No)

m. Exercising (Yes/No)

23) Assume that you have hypothetically become suspected of being a COVID-19 carrier and are recommended to be in self-isolation. How would you react to this hypothetical scenario?

a. I will accept self-isolation cooperatively & it will take priority over my personal affairs

b. I will accept self-isolation, but my personal affairs are more important

c. I will not accept self-isolation because it is only suspected and not confirmed

24) How much of your information about COVID-19 comes from television (i.e, national channels)?

a. (1 very limited ←→ 5 significant amount)

25) How much of your information about COVID-19 comes from newspapers and magazines (this includes both printed and online versions)?

a. (1 very limited ←→ 5 significant amount)

(Salimi et al., 2020)

26) How much of your information about COVID-19 comes from social media (Facebook, Instragram, etc)?
    a. (1 very limited ←→ 5 significant amount)
27) How much of your information about COVID-19 comes from friends?
    a. (1 very limited ←→ 5 significant amount)
28) How much of your information about COVID-19 comes from official health agencies (United States Public Health Service; National Institutes of Health; Centers for Disease Control and Prevention; Health Canada; Public Health Agency of Canada, etc)?
    a. (1 very limited ←→ 5 significant amount)
29) With regards to COVID-19, how much confidence do you have in information coming from television sources (i.e, national channels)?
    a. (1 very limited ←→ 5 significant amount)
30) With regards to COVID-19, how much confidence do you have in information coming from newspapers and magazines (this includes both printed and online versions)?
    a. (1 very limited ←→ 5 significant amount)
31) With regards to COVID-19, how much confidence do you have in information coming from social media (Facebook, Instragram, etc)?
    a. (1 very limited ←→ 5 significant amount)
32) With regards to COVID-19, how much confidence do you have in information coming from friends?
    a. (1 very limited ←→ 5 significant amount)
33) With regards to COVID-19, how much confidence do you have in information coming from official health agencies (United States Public Health Service; National Institutes of Health; Centers for Disease Control and Prevention; Health Canada; Public Health Agency of Canada, etc)?
    a. (1 very limited ←→ 5 significant amount)
34) In your opinion, was the public health response in your country sufficient/adequate?
